# Evolution of microbial dynamics with the introduction of real seawater portions in a low-strength feeding anammox process

**DOI:** 10.1007/s00253-020-10598-9

**Published:** 2020-04-17

**Authors:** Xiaoming Ji, Yongli Wang, Po-Heng Lee

**Affiliations:** 1 Department of Civil and Environmental Engineering, Hong Kong Polytechnic University, Hung Hom, Kowloon, Hong Kong, China; 2 Department of Civil and Environmental Engineering, Imperial College London, South Kensington Campus, London, UK

**Keywords:** Low-strength wastewater, Real seawater, *Ca. Brocadia*, Microbial community, Nitrite-oxidizing bacteria

## Abstract

The salinity effect on anammox bacteria has been widely reported; however, rare studies describe the microbial dynamics of anammox-based process response to the introduction of real seawater at mainstream conditions. In this study, an anammox process at mainstream conditions without pre-enriching anammox bacteria was shifted to the feeds of a synthetic wastewater with a portion of seawater mixture. It achieved over 0.180 kg-N/(m^3^ day) of nitrogen removal rate with an additional seawater proportion of 20% in the influent. The bacterial biodiversity was significantly increased with the increase of seawater proportions. High relative abundance of anammox bacteria (34.24–39.92%) related to *Ca. Brocadia* was enriched and acclimated to the saline environment. However, the introduction of seawater caused the enrichment of nitrite-oxidizing *Ca. Nitrospira*, which was responsible for the deterioration of nitrogen removal efficiency. Possible adaptation metabolisms in anammox bacteria and other nitrogen transforming bacteria are discussed. These results highlight the importance of microbial diversity for anammox process under the saline environments of 20% and 40% seawater composition.

## Introduction

Anaerobic ammonium-oxidizing (anammox)-based processes have attracted increasing attention due to its advantages: less oxygen requirement, no carbon addition, and less sludge production (Du et al. 2015; Lackner et al. 2014; Ma et al. 2017; Siegrist et al. 2008). Anammox process was successfully applied for high-strength wastewater treatment (Ma et al. 2016), and in recent, an increasing of studies supported its feasibility for mainstream (Agrawal et al. 2018; Laureni et al. 2016). However, with the development of coastal cities, the production of saline wastewater is increased. One of such causes is seawater intrusion into coastal freshwater aquifers. The over-extraction of groundwater resources is one of the important reasons (Li et al. 2017). Meanwhile, seawater was directly used in coastal cities due to the shortage of freshwater. For example, seawater was used to flush toilets in Hong Kong (Leung et al. 2012). Both of these two contributors will result in an introduction of seawater in mainstream wastewater (Bear et al. 1999; Li et al. 2017), increasing the complexity for biological wastewater treatment. Specially, the introduction of seawater results in an additional challenge for the mainstream anammox processes.

Previous studies explored the salinity effect on anammox bacteria through adding NaCl (Chen et al. 2014; Jin et al. 2011; Li et al. 2018; Kartal et al. 2006; Xing et al. 2015; Zhang et al. 2018). Kartal et al. (2006) reported that the dominant anammox bacteria shifted from *Ca. Brocadia* to *Ca. Kuenenia* with the increasing of salinity concentration. Previous studies also showed that *Ca. Kuenenia* could adapt to a higher salinity of 15–30 g/L NaCl/KCL; however, a salinity over 4 g/L has a great impact on Ca. Brocadia (Ali et al. 2020). It is likely that *Ca. Kuenenia* could endure a higher salinity over *Ca. Brocadia*. Apart from anammox bacteria, the microbial community structure was significantly affected by wastewater constituents (Gonzalez-Martinez et al. 2015; Liu et al. 2018). The evolution of microbial structure also provides a comprehensive understanding on the nitrogen transformation of anammox-based processes, and thus further influences nitrogen removal optimization (Vlaeminck et al. 2012). Recently, the microbial interaction in the anammox-based systems was investigated by Speth et al. (2016) and Lawson et al. (2017), indicating that microbial community is correlated to reactor performance. Obviously, the complexity in real seawater composition, such as sulfate and organics, is much more than synthetic wastewaters with only NaCl addition. For example, heterotrophic denitrifiers could benefit from the additional carbon source and might enhance the nitrogen removal efficiency through denitrification (Lotti et al. 2015). Sulfate might be metabolized by anammox bacteria or denitrifiers as electron acceptors, affecting the microbial community in return (Yang et al. 2009). Additionally, ammonium oxidizing bacteria (AOB) and nitrite oxidizing bacteria (NOB) widely detected in anammox-based systems (Pereira et al. 2017) might be affected by the introduction of real seawater as well. Although the effects of real seawater on anammox process for nitrogen-rich wastewater treatment had been reported (Qi et al. 2018), limited reports describe under saline low nitrogen loading conditions, especially for the evolution of microbial dynamics with different proportions of seawater feed.

Therefore, this study characterized the microbial evolution in anammox process for low-strength wastewater with different proportion of real seawater. A continuous flow anammox reactor was set up at room temperature without a pre-enriching high abundance of anammox bacteria. Investigation was made for the reactor performance and each corresponding microbial structure at different seawater proportions, with special attention on the anammox bacteria, AOB, and NOB. Their potential adaptation metabolisms towards seawater-based wastewater in Anammox bacteria and other nitrogen transforming bacteria were addressed.

## Material and methods

### Reactor set-up, inoculum, and operation strategy

**Fig. 1:**
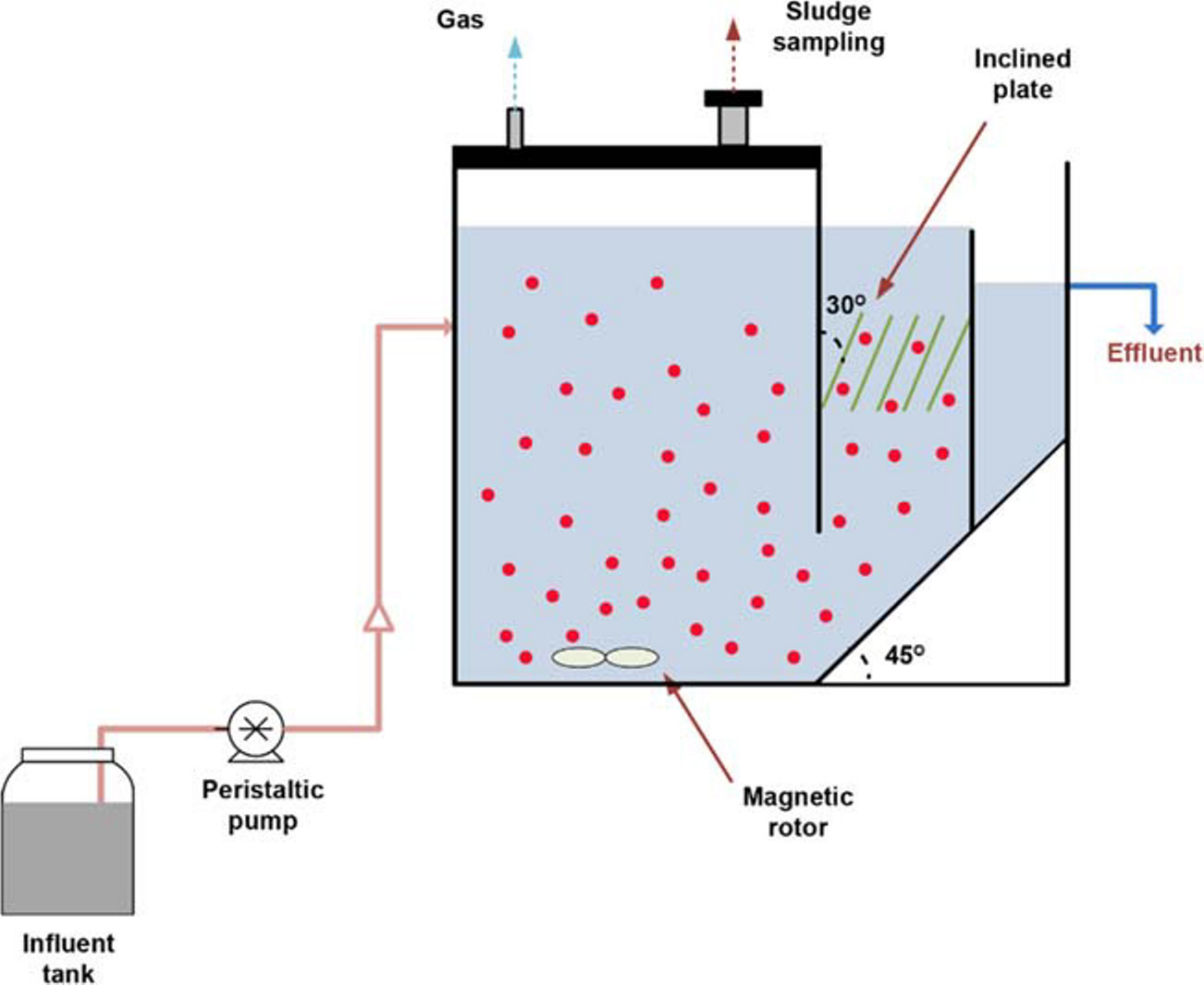
The CSTR in this study

A lab-scale anammox continuous stirred-tank reactor (CSTR) with an effective volume of 1.0 L was set up and operated at room temperature (Fig. 1). Magnetic stirring with 50 rpm was used for mixing in the reactor. Biomass was intercepted through the inclined plate installed in the sedimentation area. The reactor was inoculated with dried powdered sludge taken from a full-scale simultaneous partial nitrification, anammox, and denitrification (SNAD) reactor in Taiwan (Taiwan, China) (Wang et al. 2010). The dried powdered sludge was soaked in the buffer solution for activation before inoculum in the reactor. The buffer was made with NH_4_Cl, NaNO_2_, KHCO_3_, KH_2_PO_4_, and K_2_HPO_4_. The initial mixed liquor suspended solids (MLSS) in the CSTR was about 7600 mg/L.

The reactor was fed with a synthetic wastewater (without pre-removing dissolved oxygen) containing NH_4_Cl, NaNO_2_, KH_2_PO_4_, MgSO_4_·7H_2_O, and 1 mL/L of trace element solution as described by van de Graaf et al. (1996), and different proportions of real seawater. Real seawater was collected from the Hong Kong Victoria Harbour without any pre-treatment process, which the salinity was 3.1~3.3% at the point of collection. The characteristics of collected seawater displayed as follows: 2.12~3.31 g SO_4_^2−^ L^−1^, 0~1.8 mg BOD_5_ L^−1^, and 6.3~14.2 mg COD L^−1^. The influent concentration of ammonium and nitrite was configured as needed. The influent pH was adjusted to 8.0 without further adjustment.

### Analytical methods

The collected influent and effluent samples were filtered (0.45 μm) before analyze. The influent and effluent quality parameters of NO_3_^−^-N, NO_2_^−^-N, NH_4_^+^-N were measured according to standard methods (APHA 2005), and pH was measured using a pH meter (Shanghai Leici, China).

### Sludge sampling, DNA extraction, and 16S rRNA sequencing

The microbial sampling times were selected according to the initial and/or the steady-state performance at the stages of the introduced seawater of 0% (day 51 and 106 as AS1 and AS2, respectively), 20% (day 207 and 330 as AS3 and AS4, respectively), and 40% (day 408 as AS5). Biomass samples were obtained from the reactor by centrifugation (5 min, 10,000×*g*, 4 °C). DNA extraction was performed using FastDNA SPIN Kit for Soil (MP Biomedicals, Solon, OH) according to the manufacturer’s instruction, and the quality and concentrations were measured using NanoDrop® ND-1000 (NanoDrop Technologies, Wilmington, DE). The inoculated sludge had no anammox activity and the DNA concentration extracted from inoculated sludge was undetected.

DNA extracts were amplified by polymerase chain reaction (PCR) in the V3-V4 region of the 16S rRNA gene using primers 341F: ACTCCTACGGGAGGCAGCAG and 806R: GGACTACHVGGGTWTCTAAT, and were sequenced using the Illumina MiSeq platform (BGI, Shenzhen). The raw reads were processed using the MOTHUR software to remove chimeric and low-quality sequences (Schloss et al. 2009). The obtained high-quality sequences with an average length of 430 bp were clustered into operational taxonomic units (OTUs) with 97% similarity cutoff and assigned to the SILVA reference database. Raw 16S rRNA data obtained in this study had been deposited into NCBI Sequence Read Archive database with the accession number of PRJNA491507.

## Results

### Reactor performance

**Fig. 2:**
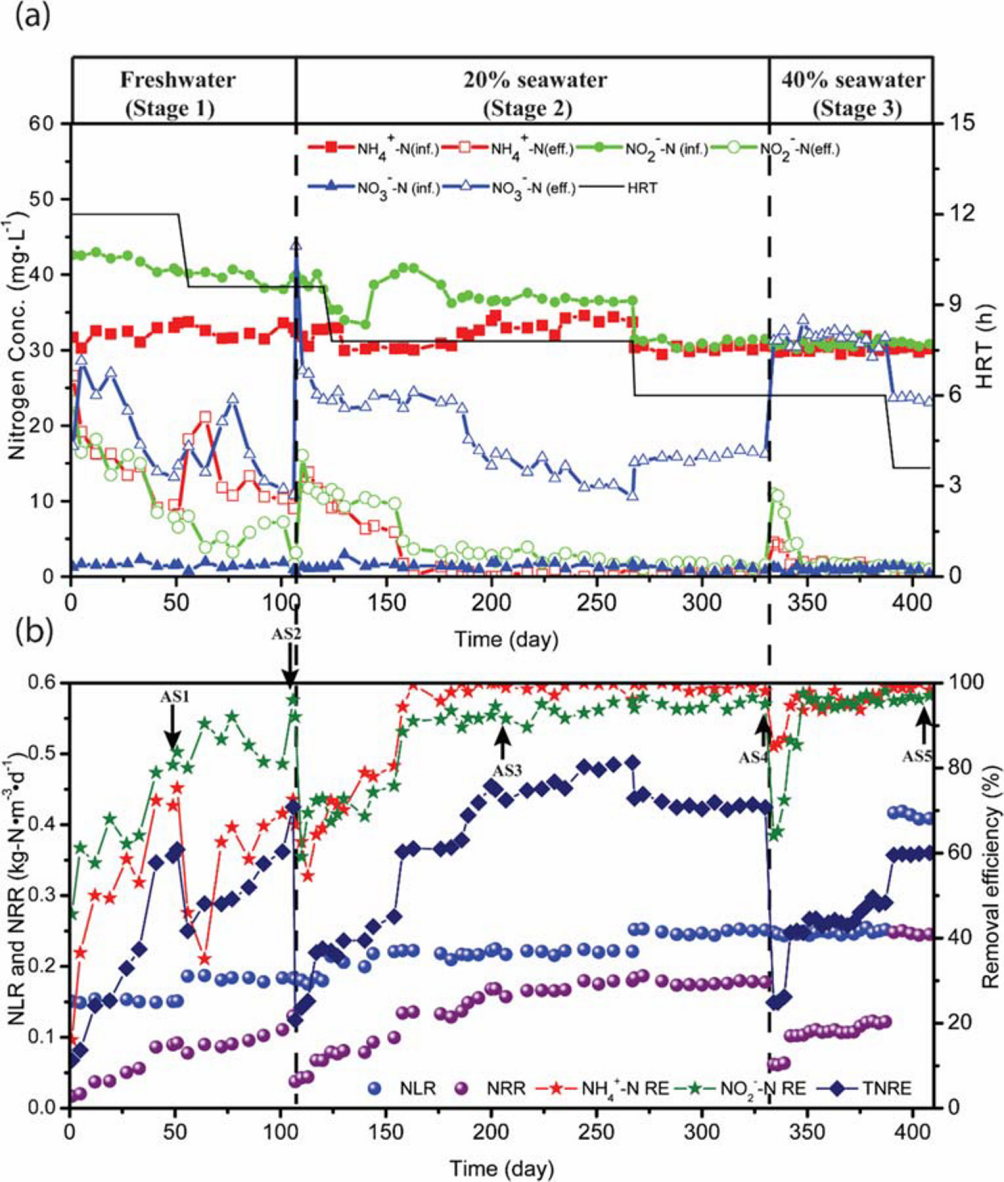
The overall performance of nitrogen removal in the CSTR during the entire operation. **a** Variation of nitrogen compounds. **b** Variation of nitrogen removal efficiency. TNRE, total nitrogen removal efficiency; NLR, nitrogen loading rate; NRR, nitrogen removal rate. The orange arrows indicate the biomass sample collection

Figure 2 shows the overall nitrogen removal performance of the CSTR. The CSTR operation was divided into three stages based on the seawater proportions of 0%, 20%, and 40% in the influent (Table 1). The total nitrogen removal efficiency (TNRE) reached around 73% at the first period, accounting for a nitrogen removal rate (NRR) of 0.130 kg-N/(m^3^ day) with a nitrogen loading rate (NLR) of 0.184 kg-N/(m^3^ day). The NH_4_^+^-N and NO_2_^−^-N removal efficiencies reached 72.60% and 95.96%, respectively. During this period, ammonium and nitrite were removed with an average NO_2_^−^-N/NH_4_^+^-N ratio of 1.55 similar to the anammox stoichiometric value of 1.32 (Fig. 2). This is different from other anammox systems inoculated with aerobic activated sludge, nitrification sludge, or anaerobic sludge, where ammonium concentration increased in the effluent, probably due to the decay of heterotrophic bacteria (Wang et al. 2009).

**Table 1:** The different operational stages throughout this study

Stage	Seawater proportion (%)	Time (d)	Influent NH_4_^+^-N concentration (mg/L)	Influent NO_2_^−^-N concentration (mg/L)	Hydraulic retention time (h)	Nitrogen loading rate (kg-N/(m^3^ day))
1	0	1–51	32.20 ± 1.04	41.78 ± 1.01	12	0.151
		52–106	32.47 ± 0.90	39.57 ± 0.94	9.6	0.184
2	20	107–120	32.05 ± 0.94	39.16 ± 0.89	9.6	0.181
		121–267	32.31 ± 1.62	36.98 ± 1.82	7.8	0.218
		268–330	30.25 ± 0.38	31.05 ± 0.48	6.0	0.249
3	40	331–387	30.29 ± 0.54	30.82 ± 0.42	6.0	0.248
		388–408	30.15 ± 0.26	30.85 ± 0.23	3.6	0.413

In the following stage (Day106 to 330), the influent wastewater contained 20% of seawater, resulting in a salinity concentration of 0.7%. As a result, the NRR dropped from 0.130 to 0.038 kg-N/(m^3^ day) sharply, representing for the TNRE from 70.85 to 20.69%, similar to the results obtained by Xing et al. (2017); however, there was almost no negative effect on the NH_4_^+^-N and NO_2_^−^-N removal efficiencies. Interestingly, the increased effluent nitrate was the main reason for the decrease of TNRE. On day 118, HRT was further reduced to 7.8 h, resulting in an increase of NLR from 0.180 kg-N/(m^3^ day) to 0.214 kg-N/(m^3^ day), even though the reactor performance was not affected and over 80% of TNRE was achieved on day 254, representing for the NRR of around 0.180 kg-N/(m^3^ day). It showed that both of NH_4_^+^-N and NO_2_^−^-N were almost removed with the effluent NO_3_^−^-N of 10.60 mg/L produced. Though this operational adjustment further into the HRT of 6.0 h resulted in the TNRE decreasing from 81.27 to 70.74%, little impact on the NRR was observed.

In the third stage (day 331 to 404), the seawater proportion was adjusted to 40%, resulting in the influent saline level of 1.5%. Similar to the former stage, the TNRE dropped from 70.74 to 24.86%, representing for the NRR from 0.178 to 0.062 kg-N/(m^3^ day), at the point of the increase of seawater proportion. Both of the NH_4_^+^-N and NO_2_^−^-N removal efficiencies decreased, corresponding to the decrease of NRR from 0.178 kg-N/(m^3^ day) to 0.062 kg-N/(m^3^ day). Moreover, the addition of seawater proportion yielded more negative impact on the NO_2_^−^-N removal efficiency (from 95.04 to 64.12%) compared with that on the NH_4_^+^-N removal efficiency (from 98.08 to 85.05%). However, the effluent NO_3_^−^-N increased from 16.25 to 31.24 mg/L. The reactor reached at steady state on day 384 with a TNRE of 48.36%, accounting for 0.122 kg-N/(m^3^ day) of NRR. It was noteworthy that both of the NH_4_^+^-N and NO_2_^−^-N removal efficiencies reached more than 98%, and nitrate was the most dominant residual nitrogen species (up to 23.09 mg/L). Furthermore, a shorter HRT (3.6 h) was applied. Interestingly, the TNRE was improved to 60.03% at the end of the study. This achieved 0.245 kg-N/(m^3^ day) of NRR with the effluent NO_3_^−^-N of 23.09 mg/L.

### The overall shifts of microbial community

#### Microbial community diversity

**Fig. 3:**
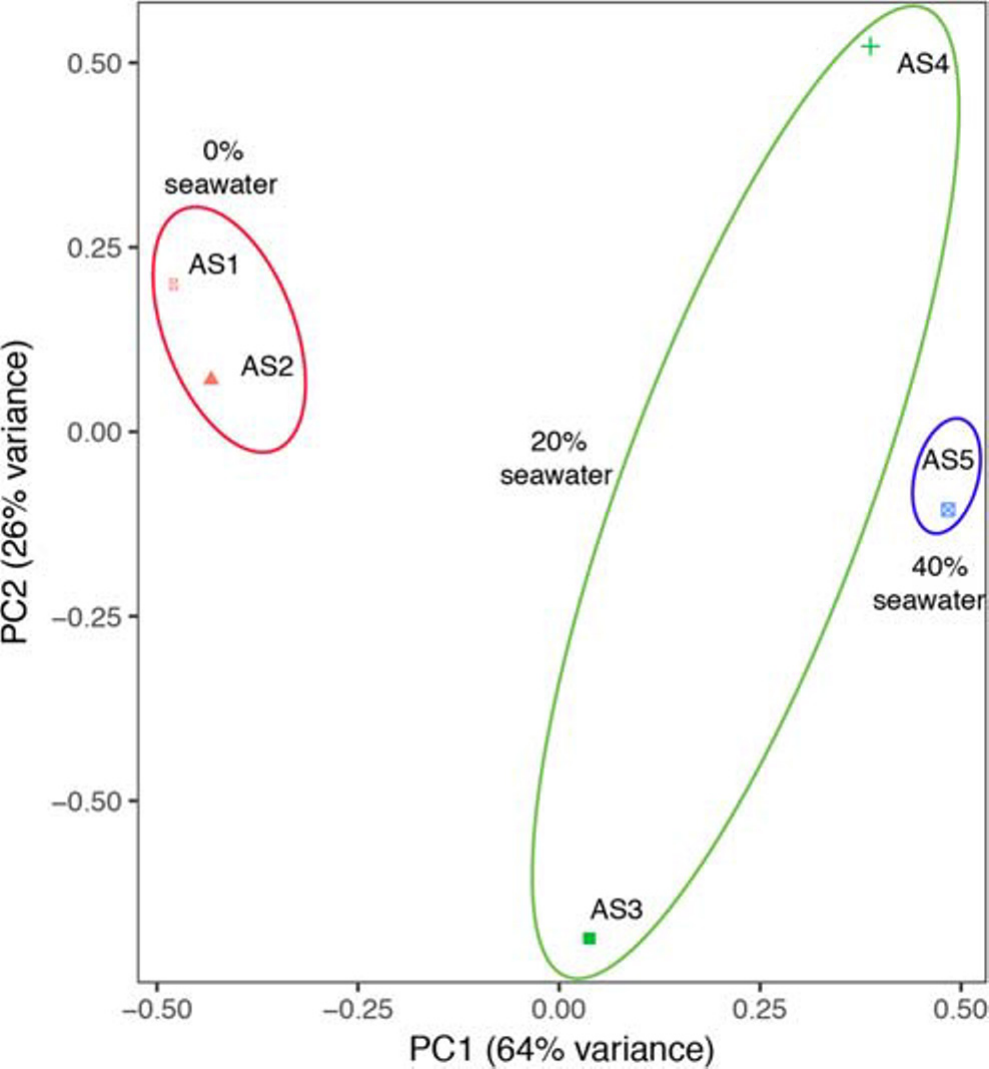
Principal components analysis (PCA) of the microbial community using dominant OTU clusters (relative abundance > 1%)

**Fig. 4:**
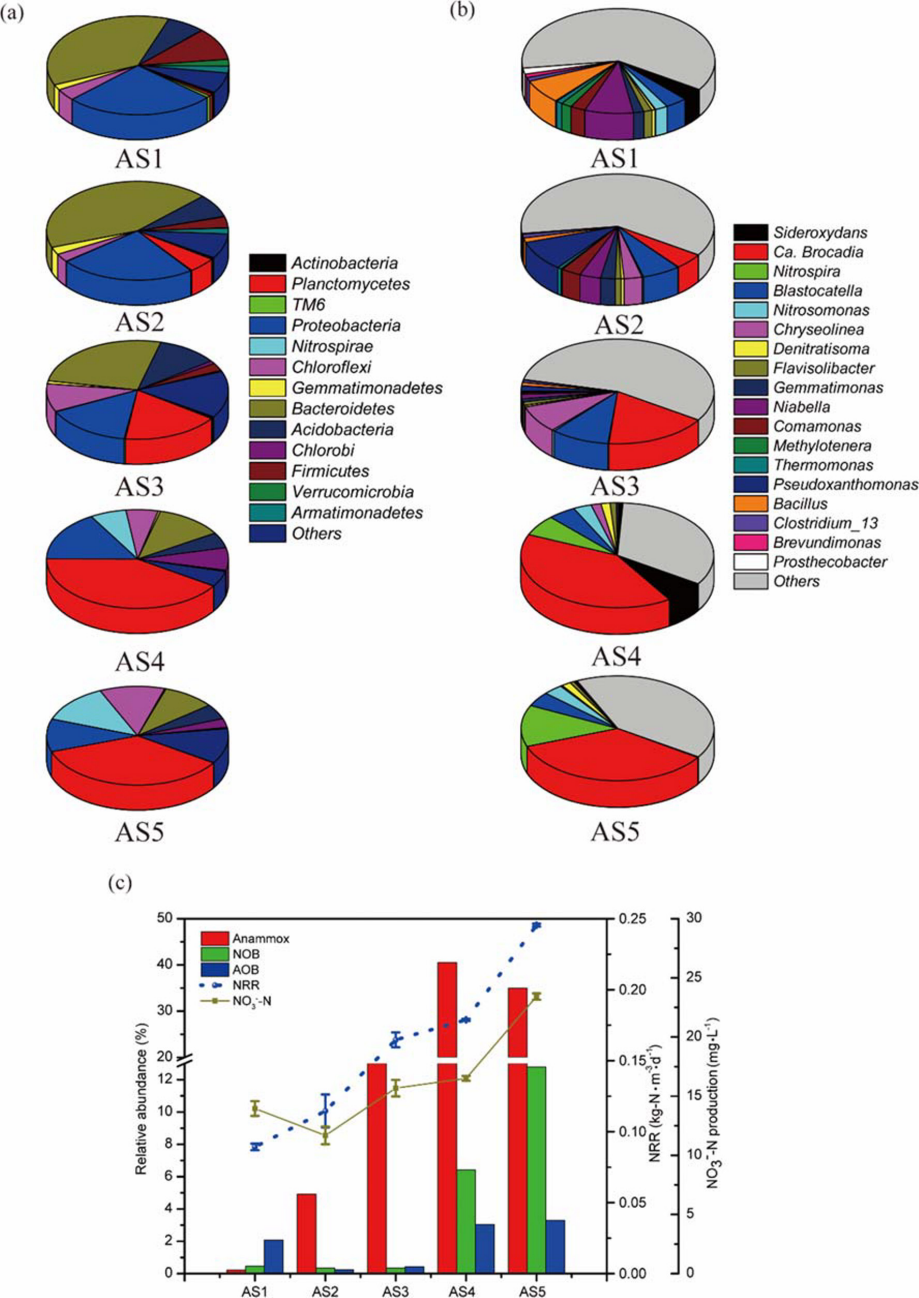
Relative abundance of major microbial community at the phylum (**a**) and genus (**b**) level by 16S rRNA sequencing. **c** The relationship between the reactor performance (NO_3_^−^-N concentration and NRR) and the dominant species (anammox, AOB, and NOB). AS1, day 51; AS2, day 106; AS3, day 207; AS4, day 330; AS5, day 408

In this study, 16S rRNA sequencing was performed for the microbial community in the CSTR over to the operational period (5 samples). The sequencing depth of all samples was more than 43,898 reads, representing over 2545 OTU clusters (Table 2). The diversity of the microbial community in response to the shift of seawater proportions was evaluated, and the results were summarized in Table 2. During the first stage, the microbial diversity decreased significantly even at an HRT as short as 7.8 h applied. Similar phenomena were obtained during the second stage that the microbial diversity decreased when the HRT was reduced to 6.0 h. This result suggested that the microbial diversity decreased with the operational time under the same seawater proportion. Additionally, note that the microbial diversity increased significantly after 20% of seawater was added to the influent. Furthermore, the microbial diversity increased when the influent of the CSTR contained 40% of seawater, while the effect on the increasing of microbial diversity was insignificant compared with feeding 20% seawater influent. It should be noted that comparing with sample AS1, the microbial diversity of AS2, AS3, AS4, and AS5 were much less, though the various seawater proportion feed was fed. Furthermore, the differences of microbial community structures over time and, with respect to different seawater proportions, were analyzed via principal components analysis (PCA) method (Fig. 3). Significant differences on microbial community structures among AS3, AS4, and AS5 were observed comparing with AS1 and AS2, while the microbial community structures of AS1 and AS2 were similar. The significant difference between AS3 and AS4 mainly caused by the enrichment of anammox bacteria since its relative abundance increased from 17.31 to 40.83% (Fig. 4b). The community structures of AS4 and AS5 were obviously different though the relative abundance of anammox bacteria is similar (Fig. 4b). Collectively, these observations indicate that the additional seawater proportion plays an important role on the diversity and structure of the microbial community.

**Table 2:** Diversity of microbial communities in different samples

Sample	Total number of sequences	OTUs	InvSimpson	Shannon	Chao	Ace	Coverage
AS1	90,609	4407	19.21	4.22	9394.61	22,008.61	0.96
AS2	99,517	3225	5.76	3.17	5838.02	12,097.02	0.97
AS3	86,969	2642	10.50	3.36	5601.31	12,088.63	0.98
AS4	75,834	3499	5.07	3.15	9897.05	24,674.50	0.96
AS5	43,898	2545	6.97	3.47	8816.28	20,324.74	0.96

#### Microbial community structure

The taxonomic results showed that the microbial community belonged to *Planctomycetes*, *Proteobacteria*, *Bacteroidetes, Acidobacteria, Chloroflexi, Firmicutes*, and *Nitrospirae* on phylum level, accounting for over 82.51% of total microbes in all samples. The relative abundance of *Planctomycetes* increased to 40.84% on day 330 in the CSTR though the seawater proportion increase from 0 to 20% (Fig. 4a). Nonetheless, its relative abundance decreased slightly (35.44%), when the seawater proportion was increased to 40%. *Bacteroidetes* was enriched in the freshwater feed, accounting for 43.20% of total microbial community on day 106; however, different from *Planctomycetes,* its relative abundance decreased significantly when seawater was introduced. The relative abundance of *Chloroflexi* increased from 2.70 to 11.60% with the freshwater and seawater addition feed. A negative impact of the seawater addition in the feed on *Proteobacteria* was observed since its relative abundance decreased from 24.29 to 10.73%. Likewise, with the seawater addition in the feed, the relative abundance of *Acidobacteria* also decreased from 7.49 to 4.90%. Furthermore, *Firmicutes* was reduced over time; however, *Nitrospirae* became one of the dominant microbes, accounting for a relative abundance of 12.79%. These results suggested that the introduction of seawater proportion affected the microbial communities in the anammox-based CSTR.

At the genus level, significant abundance differences had been observed. For example, from AS1 to AS5, the relative abundance of *Ca. Brocadia*, *Nitrospira*, *Nitrosomonas*, and *Denitratisoma* became the dominant genus. Conversely, *Niabella*, *Bacillus*, and *Comamonas* were under detected limit. Specially, among all of anammox bacteria, *Ca. Brocadia* increased from 0.22 to 35.00% with increasing seawater proportions, and it became the most dominant genus at the end of the study (Fig. 4b). Notably, *Ca. Brocadia* accounted for over 99% of *Planctomycetes* in all samples. AOB affiliated with *Nitrosomonas* genus (3.29%) was enriched in AS5. Interestingly, although the relative abundance of *Nitrosomonas* decreased to 0.25% when the CSTR was fed with the freshwater feed, it increased along with increasing proportions of seawater. It should be noted that NOB affiliated with *Nitrospira* genus enriched with a relative abundance of 12.79% in AS5 when the seawater proportion was shifted to 40%. Additionally, marine nitrite oxidizer identified to *Nitrospina* genus was detected in AS5, accounting for a relative abundance of 0.56%. The accumulated NOB due to the additional seawater resulted was reflective of high nitrate formation in the effluent (Fig. 4c). In addition to nitrifying and anammox bacteria, *Denitratisoma* (1.55%) was fostered in the CSTR at the end of the study. Moreover, no obvious differences in the relative abundance of *Denitratisoma* between AS2 and AS3, even between AS4 and AS5, though the seawater proportion was increased.

#### Anammox and nitrifying bacteria

**Fig. 5:**
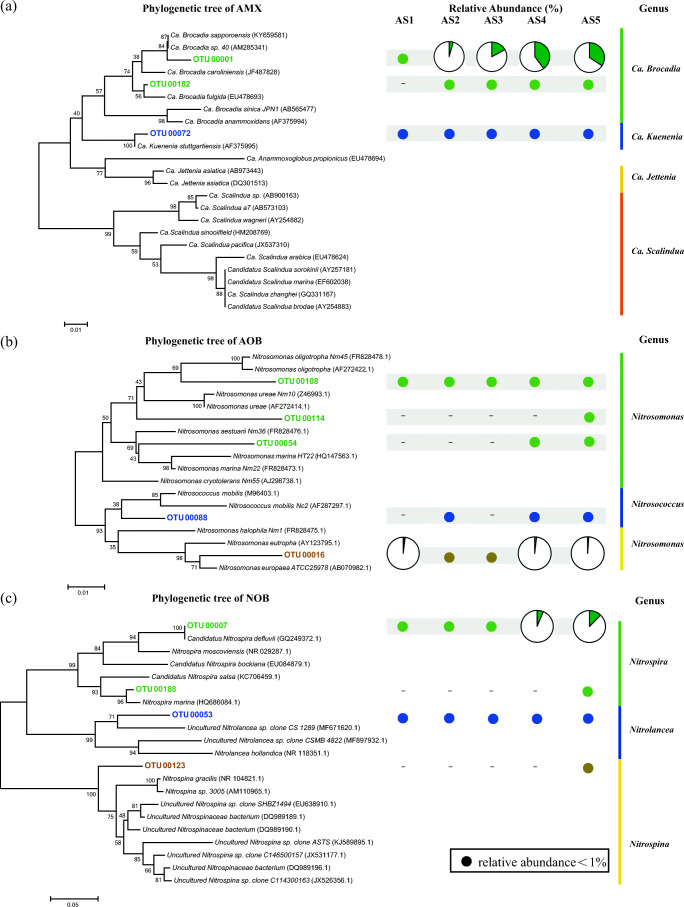
Neighbor-joining phylogenetic trees of major OTU sequences (relative abundance > 0.1%) related anammox (**a**), AOB (**b**), and NOB (**c**) in the CSTR. The color dots represent relative abundance < 1%. The pie charts represent the relative abundances of relevant OTU sequences. The trees based on Jukes-Cantor distance was constructed using Mega 7.0 with a bootstrap value of 1000

Three OTU clusters, five OTU clusters, and four OTU clusters for anammox bacteria, AOB, and NOB with relative abundance over 0.1% were selected, respectively, and further aligned with reference to construct neighbor-joining phylogenetic trees (Fig. 5). In the present study, the dominant anammox genera were not changed with the increase of seawater proportion up to 40%. *Ca. Brocadia* and *Ca. Kuenenia* were the only two anammox bacteria detected, which were consistent with the finding from Kuenen (2008). However, their relative abundance had distinctly varied due to the feed shift of seawater proportion. Taxonomic results revealed that the biodiversity of anammox bacteria was reduced significantly, because the dominant anammox bacteria (OTU00001) closed to *Ca. Brocadia* (98%) were enriched (from 0.17 to 34.24%), accounting for over 99% of all anammox bacteria in AS 2–5 (Fig. 4a). Another two anammox clusters (OTU00072 and OTU00182) identified as *Ca. Kuenenia* (99%) and *Ca. Brocadia* (98%) were also observed, respectively. Different from the genus *Ca. Brocadia*, the relative abundance of anammox cluster OTU00072, similar to *Ca. Kuenenia*, decreased with the increasing portioned of seawater, which agrees with the finding by Wu et al. (2019). Additionally, it should be pointed out that no *Ca. Scalindua* with better adaptation in marine environment was detected.

Different from anammox bacteria, the biodiversity of AOB and NOB increased obviously along the increase of seawater proportions. The shift of seawater proportions affects not only AOB and NOB but also their relative abundance. The phylogenetic composition of AOB and NOB was also investigated (Fig. 5b and c). In total, five OTU clusters affiliated to *Nitrosomonas* and *Nitrosococcus* were detected in AS5; however, only two clusters affiliated to *Nitrosomonas* were observed in AS1. Indeed, the AOB populations were almost dominated by the same cluster (OTU00016), except in AS3 (Fig. 5b). There was almost no shift of the dominant AOB during the low strength wastewater treatment with different seawater proportions up to 40%. Previous studies revealed that several AOBs, such as *Nitrosomonas europaea*, could outcompete others from the selective environment due to their rapidly growth rate (Ahn et al. 2008; Reino et al. 2016).The phylogenetic analysis reveals that AOB affiliated with genus *Nitrosococcus* appeared when the CTSR was altered to the saline environment, accounting for 0.8% of total reads in AS5. These detected genus *Nitrosococcus* might belong to marine AOB since some of them were obligate halophilic and found in the marine environment (Ahn et al. 2008).

Similar situation as AOB had also been observed for NOB, whose OTU clusters increased from two to four. Two clusters (OTU00007 and OTU00053) affiliated with *Nitrospira* and *Nirolance* genera were detected in all sludge samples, respectively (Fig. 5c). Two unique clusters related to genus *Nitrospira* and *Nitrospina* were only observed in AS5, suggesting the biodiversity of NOB increased suddenly when the seawater proportion was raised to 40%. Phylogenetic analysis revealed that cluster OTU00007, similar to *Nitrospira* (100%), turned into the most dominant NOB (12.33% of total reads) with the increase of seawater proportion. Note that among genus *Nitrospira*, a few of species, such as *Nitrospira marina,* are capable of inhabiting in marine environments with its best growth under mixotrophic conditions (Watson et al. 1986). Similar to *Nitrospira marina*, the *Nitrospina* genus was exclusively found in marine conditions (Ngugi et al. 2016). These results reveal that marine NOB might be accumulated in anammox-based process due to the induction of seawater.

## Discussion

The anammox activity was affected by the introduction of seawater obviously since the NRR dropped when the seawater feed (20% seawater portion) was introduced (Fig. 2b). However, the TNRE recovered back to 80% under a low saline condition (20% seawater addition feed), indicating the anammox activity could recover through domestication. For the long-term operation, *Ca. Brocadia* genus could adapt to the salinity environment with 40% of seawater, as was also revealed by Dapena-Mora et al. (2007) and Kartal et al. (2006). The reactor performance responses to the salinity shock could be divided into the sensitive, interim stable, and recovery stage (Ma et al. 2012). In this study, the recovery period of the salinity shock at 20% saline seawater addition feed (the system performance back to 80% of TNRE) is around 150 days when the seawater proportion was shifted to 20%. The recovery time was longer than the results from Ma et al. (2012). The relative abundance of anammox bacteria and each corresponding saline adaptivity may affect the recovery time, since each anammox bacterium responses differently both in the salinity shock period followed by the adaptation one (Tang et al. 2011; Wu et al. 2019). While the seawater addition feed (20%) was first introduced, the relative abundance of anammox bacteria was only 4.91% with no acclimation to saline condition previously, resulting in its weak response to salinity. Unlike the first stage of salinity shock, the respondence time was shortened at the seawater addition feed from 20 to 40%, probably due to the relative high abundance of anammox bacteria (40.52%) already in the CSTR (Fig. 2). This might be associated to its long-term adaptivity. As for NOB, the effluent nitrate correspondingly increased with the salinity shock, indicating that NOB was more adaptable to the salinity shock compared with anammox bacteria no matter that its relative abundance was less than anammox bacteria. Accordingly, with the high production of nitrate, TNRE was reduced along with the increase of seawater proportion (Fig. 2b). However, 80% of TNRE was achieved in the anammox CSTR with the low strength seawater-based wastewater (20% of seawater portion) from enriching and domesticating freshwater anammox bacteria synchronously. This supports a broad application of anammox-based processes in dealing with seawater-intruded sewage, saline sewage, and seafood processing wastewaters that generally consist about 20% of seawater (Leung et al. 2012).

An interesting observation is that the introduction of seawater induced the succession of heterotrophs during the start-up in the low-strength seawater anammox-based system. Certainly, we observed that seawater had a positive impaction on the relative abundance of *Chloroflexi*, albeit different from Gonzalez-Silva et al. (2017), in which the relative abundance of *Chloroflexi* decreased in anammox consortia when NaCl was added. Generally, the phyla of *Chloroflexi* has been found to be interacted with anammox bacteria through organic matters exchange such as extracellular polymeric substances (EPS) or Vitamin B12 in various reactors (Lawson et al. 2017). Distinguishably, in this present CSTR system, the additional seawater introduces not only sodium but also sulfate, organic matters etc. These might be influential in the metabolism of *Chloroflexi.* For instance, *Chloroflexi* possibly benefits from the introduced organic matters and ions for its energy source and proton/sodium gradient of ATP formation in seawater, respectively. Moreover, the presence of sulfate is the potential electron acceptor for denitrification mediated by *Chloroflexi*. Similar to *Chloroflexi*, the presence of *Acidobacteria*, utilizing various organic matters (e.g., degrade xylan, chitin, cellulose, and hemicellulose), has also been reported in various anammox systems (Gonzalez-Martinez et al. 2015; Pereira et al. 2017; Costa et al. 2014) but an adverse effect on salinity (Zheng et al. 2017), suggesting its overlooked role in anammox consortia. *Bacteroidetes* is one of the common phyla in anammox systems, possibly interacting with other microorganisms by various organic matters (Costa et al. 2014). In this study, the relative abundance of *Bacteroidetes* decreased significantly due to the introduction of seawater, which contrasts with the finding by the addition of NaCl as the saline source (Gonzalez-Silva et al. 2017). The salinity may not play a key element for *Bacteroidetes*, due to its high resistance to salt. It is possible that *Bacteroidetes* cannot compete for substrates with *Chloroflexi* under such environments.

In the present CSTR, two anammox genera (*Ca. Brocadia* and *Ca. Kuenenia*) were enriched during the freshwater treatment period. Previous studies revealed that *Ca. Kuenenia* was the dominant anammox bacteria treating saline wastewater (Gonzalez-Silva et al. 2017; Kartal et al. 2006) but was not sustainable with the increase of seawater proportion in this study. Conversely, high relative abundance (34.24%) of anammox bacteria (OTU 00001) close to *Ca. Brocadia* was enriched (Fig. 5a) and dominated the whole process, which was different from the findings of the dominance of *Ca. Brocadia fulgida* or *Ca. Kuenenia stuttgartiensis* through adding NaCl as salinity by Gonzalez-Silva et al. (2017). Based on a previosus kinetic study (Zhang et al. 2017), one hypothesis is a higher growth rate of *Ca. Brocadia* sp. over Ca. *Kuenenia stuttgartiensis*, suggesting that the former has a higher substrate affinity over the latter under the limited substrate supply (low-strength wastewater). Additionally, *Ca. Brocadia* was proposed favorable in floc; *Ca. Kuenenia* likely intended to present in biofilm or granular (Guo et al. 2016), thereby outcompeting through a synergetic association with *Chloroflexi* and others. As aforementioned, the high relative abundance of *Ca. Brocadia* (OTU 00001) over *Ca. Kuenenia* was obtained during the freshwater treatment period. Recently, de Almeida et al. (2016) proposed that *Ca. Kuenenia stuttgartiensis* utilized the sodium-motive force (*smf*) for ATP formation. Specifically, the additional seawater would drive the enzyme-encoding sodium pumping NADH: quinone oxidoreductase (sodium-NQR) and Na^+^-ATPases to transport sodium ion into cell for *smf* generation, rather than proton motive force (Paparoditis et al. 2014). Next, the enzyme-encoding Na^+^-translocating (RNF) is activated by the *smf* and pump Na^+^ out with reduction of ferredoxin for carbon metabolism (de Almeida et al. 2016). Whether the dominant *Ca. Brocadia* could apply the *smf* is an intriguing question deserving for further exploration.

We observed that the relative abundance and biodiversity of nitrifying bacteria (AOB and NOB) increased with the increase of seawater proportion. Taking the reactor performance and taxonomic results together, the relative abundance of dominant NOB (OTU0007) affiliated to *Ca. Nitrospira* increased along with the increase of seawater proportion. It is possible that the dominant NOB could be tolerant to the salinity (Wang et al. 2017), but the opposite point of views was also reported elsewhere (Bassin et al. 2011; Cui et al. 2009; Hunik et al. 1993; Pronk et al. 2014; Vredenbregt et al. 1997). One is that the simple organic carbons contained in the real seawater may serve as electron donors for the metabolic flexibility of NOB, such as *Ca. Nitrospira defluvii* (Daims et al. 2016). Simultaneously, some NOB had been speculated to be non-obligate aerobic speices (Füssel et al. 2012). A previous study reported that *N. moscoviensis* using O_2_ as the electron acceptor could reduce nitrate reversely with formate as an electron donor, and further to re-oxide the produced nitrite back to nitrate (Koch et al. 2015). Another possibility is that sodium ion in seawater may also induce sodium-ATPase for energy harvest as is similar to *Ca. Kuenenia stuttgartiensis*. However, the increase of seawater proportion in this study resulted in the dominance of NOB and the accumulation of nitrate.

In conclusion, the mainstream seawater-based feed was introduced to the anammox CSTR in this study. The bacterial diversity and structure in the CSTR were significantly affected by the feed proportion of the real seawater. High relative abundance of anammox bacteria (34.24–39.92%) related to *Ca. Brocadia* (98%) was enriched. The introduction of seawater, especially for 40% seawater protion, caused the appearance of marine nitrifiers, which aggravated the deterioration of reactor performance. It also suggests the effect on the microbial community in anammox process by using the real seawater is different from that only using NaCl, providing a guidance for practical engineering applications.
